# On the Person-Place Interaction and Its Relationship with the Responses/Outcomes of Listeners of Urban Soundscape (Compared Cases of Lisbon and Bogotá): Contextual and Semiotic Aspects

**DOI:** 10.3390/ijerph16040551

**Published:** 2019-02-14

**Authors:** Luis Hermida, Ignacio Pavón, Antonio Carlos Lobo Soares, J. Luis Bento-Coelho

**Affiliations:** 1 Department of Mechanical Engineering, Escuela Técnica Superior de Ingenieros Industriales, Universidad Politécnica de Madrid, 28006 Madrid, Spain; ignacio.pavon@upm.es; 2 Faculty of Engineering, Universidad de San Buenaventura-Bogotá, 110141 Bogotá, Colombia; 3 Instituto Superior Técnico, University of Lisbon, 1049-001 Lisboa, Portugal; lobosoares@hotmail.com (A.C.L.S.); bcoelho@ist.utl.pt (J.L.B.-C.); 4 Ministério da Ciência, Tecnologia, Inovações e Comunicações, Museu Paraense Emílio Goeldi, 66.040-170 Belém, Pará, Brazil

**Keywords:** urban environments, soundscape, semiosis model

## Abstract

Design, planning, and management of the urban soundscape require various interacting fields of knowledge given the fact that it is the human person that experiences and provides meaning to the urban places and their acoustic environments. The process of environmental perception involves contextual information that conditions people’s responses and outcomes through the relationship between the variables Person, Activity, and Place. This research focuses on the interaction between Person and Place and its impact on responses and outcomes from listeners with different geographical origin and background. Laboratory studies were conducted in the cities of Lisbon (Portugal) and Bogotá (Colombia), where local listeners were introduced to known and unknown acoustic environments. Sound data recorded in the two cities allowed comparison of responses and outcomes of the listeners according to the Person-Place Interaction, leading to different meanings depending on the contextual variables. The results clearly show a relationship between site, acoustic environment, soundscape, Person-Place Interaction, and meaning of the place. This information can be useful for urban technicians and designers dealing with planning and management of urban soundscapes.

## 1. Introduction

The traditional approach to environmental acoustics management has mainly revolved around the control of sound energy levels, according to criteria established by governmental environmental agencies in each country. Nonetheless, considering the impact of the acoustics environments on people’s health and life quality [1,2,3,4,5], and the need to promote and preserve quiet areas [6,7], the conventional approach has had to broaden its scope and to redirect the focus where the human being is considered the center [8,9,10,11,12,13,14,15], which has led to the strengthening of the soundscape concept.

Soundscape only exist through human perception, therefore its study, evaluation, and design require an interdisciplinary work, where in addition to dealing with aspects of characterization of the acoustic environment, it is also necessary to analyze the way human beings perceive and understand their acoustic environment [1,16,17]. Therefore, in order to approach the soundscape concept, research has been conducted in different lines, such as the description and classification of different taxonomic aspects of acoustic environments [1,18,19] and to determine acoustic descriptors and perceptual models that may prove useful in the assessment of soundscapes [20,21,22,23,24,25].

Regarding the perceptual processes, they are mediated by sociocultural and psychological aspects which in turn are conditioned by context [26,27]. In the conceptual framework proposed in the standard ISO 12913-1, the context influences the sound sources, the auditory sensation, and the cognitive process, thereby conditioning the person’s responses and outcomes [28,29]. Given the preceding, it can be determined that the design, management, and planning of soundscape not only comprises aspects of design and engineering, but also encompasses a different kind of view based on ecological perception, environmental experience, and communication.

Under an ecological approach, human beings have a direct relation with their environment [30,31], where they find a varied affordance of stimuli that trigger a high number of physiological and psychological aspects, allowing them to collect and obtain information about their surrounding environment. This information enables experiencing sensations or emotions and consequently determines a response [32]. Likewise, a relation can be established between the social psychology approach and the perceptual model of soundscape in the standard ISO 12913-1, due to the fact that responses and outcomes represent, respectively, attitudes (referring to the degree to which people tend to judge aspects of reality) and behaviors (considered the set of responses or decisions resulting from the relation between the people and the environment) of the human being [33,34]. In other words, during one’s immersion in an acoustic environment, human attitudes are generated (e.g., how quiet, pleasant or annoying is the place) and from there actions follow (e.g., perform a specific activity or decide to leave the place).

Regarding the process of environmental experience, Herranz-Pascual et al. have been developing work in the soundscape field following this approach, proposing a model of environmental experience for studying soundscape, based in the psychological process of environmental experience [32,34,35,36]. This model comprises three fundamental aspects: *Person*, *Activity,* and *Place*. These three elements generate four entities that can influence the experience of the acoustic environment: *Person*, *Activity*, *Place* and *Person-Place Interaction*. Nevertheless, taking the model of environmental experience of soundscapes to an effective practice of evaluation and design involve many variables (e.g., security, climate, previous experiences, place function, urban aspects, visual and olfactory stimuli, etc.), so it is necessary to determine which variables are the most influential in the environmental sound experience, as well as establishing processes and procedures for this type of analysis. Currently, most studies in this field focus on the influence of alternative stimuli to sound [35,37,38,39,40,41,42,43], detecting that one of the most influential stimulus in the perception of the acoustic environment is the vision. With regard to the study of contextual elements related to the *Person-Place Interaction*, reference is made in the cross-national comparison in assessment of urban park soundscapes, in the analysis and assessment of soundscapes in commonly used sightseeing sites (comparison between tourists and non-tourists), as well as comparison between responses of experts and non-experts [27,44,45,46].

Conversely, according to Blauert “the behavior of human beings is not guided directly by the acoustical signals that we provide them with, e.g., like a reflex, but rather by the “meaning” which is transferred via these signals” [47], which implies that acoustic environments also carry an important informational load when they are considered as “carrier” of meaning. In semiotics, the science of signs and languages, a sign can be defined as the mental representation processed as a reference of something else [48,49,50]. The study of urban environments under this approach has allowed analyzing the impact of different kind of information (mainly visual) in the identity of the place and the acquired meaning depending on the context of the person [51,52,53,54]. Now consider the question: What is the meaning that designers, administrators, and urban planners want to convey to people from acoustic environments? Note that the meaning depends on the repertoire and context of the listener, thus the study of contextual variables in the formation of meaning of acoustic environments becomes necessary.

Considering that: (1) A direct interaction between the person and the acoustic environment generates an *environmental experience*, when responses and outputs are mediated by the context, and (2) that acoustic environments can be analyzed as carrier of meaning that also depends on the context, this research seeks to deepen in the study of the relationship between the responses (attitudes) and outcomes (behaviors) of the listeners and the contextual element *Person-Place Interaction*, as well as to analyze the importance of semiotic aspects in the design, management, and assessment process of soundscapes. For this purpose, laboratory tests were developed in the cities of Lisbon (Portugal) and Bogotá (Colombia), where data on acoustic environments were presented to two groups of listeners from their own city and from a city that they haven’t previously experienced. Differences were found in the *outcomes* according to the origin places of the listeners, allowing to establish a relationship between the use of places, their meaning (or lack of it) and the soundscapes.

## 2. Method

### 2.1. Sites of Study

Considering that the objective of this work was not to compare the acoustic environments of two different cities, but to analyze the responses and outcomes of people according to the variable Place-Person and its impact on the formation of meaning, eight places were chosen from the cities of Bogotá and Lisbon. The site selection was aimed at capturing the city’s diverse acoustic environments, including high-speed road, city center, square with a water source, urban parks, and quiet areas.

The sites in Lisbon consisted of urban public places of great recognition among the inhabitants:

- Jardim da Estrela (LJES),
- Jardim da Fundação Calouste Gulbenkian (LJFG),
- Jardim do Príncipe Real (LJPR).

The selected sites in Bogotá were:

- Autopista norte estación Alcalá (BOAPA),
- Calle 19 con Cra. 7 (BOC197),
- Fuente de agua edificio Tequendama (BOFAET),
- Plaza de Lourdes (BOPL),
- Simón Bolívar park (BOPSB).

**Figure 1:**
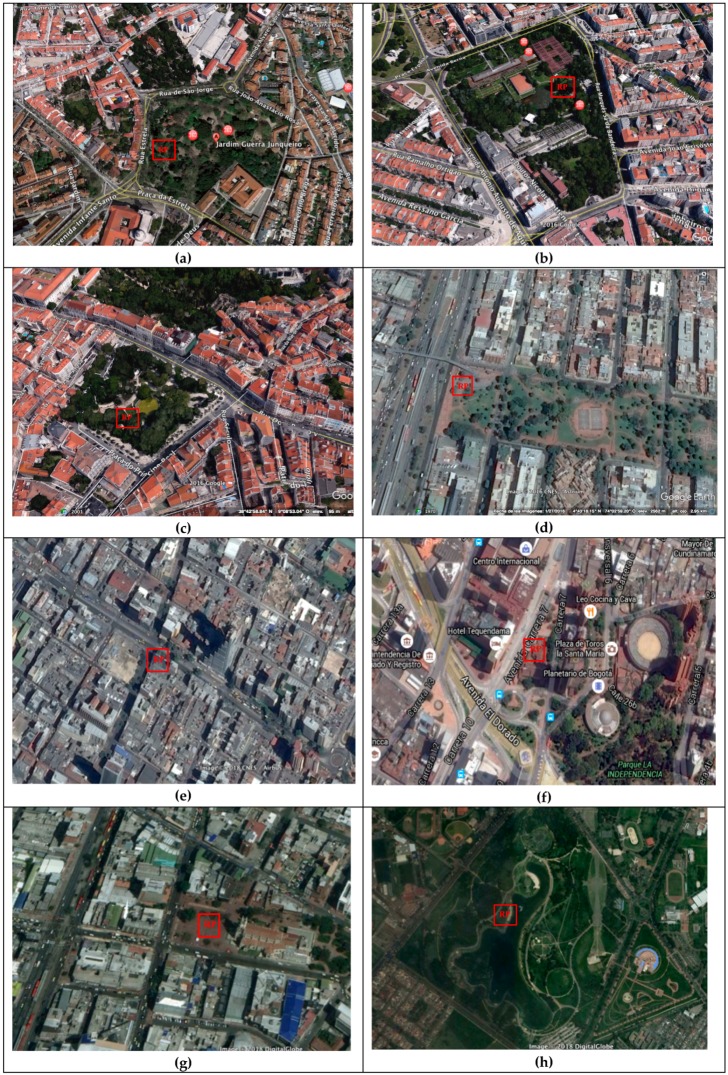
Urban Spaces in Lisbon and Bogotá cities. (**a**) Jardim da Estrela (LJES); (**b**) Jardim da Fundação Calouste Gulbenkian (LJFG); (**c**) Jardim do Príncipe Real (LJPR); (**d**) Autopista norte estación Alcalá (BOAPA); (**e**) Calle 19 con Cra. 7 (BOC197); (**f**) Fuente de agua edificio Tequendama (BOFAET); (**g**) Plaza de Lourdes (BOPL); (**h**) Simón Bolívar park (BOPSB).

The Lisbon sites have different uses, characteristics, and locations, with urban areas of 7.5 ha (LJFG), 4.6 ha (LJES) and 1.15 ha (LJPR). They feature water fountains, diverse vegetation, children playgrounds, good infrastructure, cleanliness, security, and are located in privileged areas of the city, surrounded by buildings of aligned storefronts with a maximum of six floor-to-ceiling-height, where noise (traffic, road, and aerial) can be perceived by the inhabitants. Like the Lisbon sites, the Bogotá sites offer a great diversity of acoustic environments, presented the most important roadway of the city (BOAPA); the downtown area (with vehicular traffic and street vendors) (BOC197); a square with a water fountain (BOFAET); one of the most representative squares of the city of Bogotá (BOPL) and one of the largest urban parks in South America with 113 ha (BOPSB). Figure 1a–h depict the urban spaces in Lisbon and Bogotá.

As the analysis of the human perception of acoustic environments is required for soundscape studies, binaural recordings were made. This type of recordings allow an image of the spatial distribution of the sound sources, and generate sound immersion in the listeners [55,56,57,58]. While each recording is unique and unrepeatable due to the renewable temporal characteristics of the acoustic environments, it was sought that each recording would present to the listeners sounds of the most common and representative activities in each place. For this purpose, prior to the recording process, the places under study were visited, to gather *in-situ* information on the usual on-going activities. These data were used in the process of selection and edition of the audio fragments presented to the listeners in the laboratory test.

A portable binaural recording system was used with the purpose of not interfering with the free development of everyday activities or to draw the passers-by’s attention to foreign elements oblivious to their daily lives (e.g., binaural heads). The recordings were made in each place at fixed points parallel to the main sound sources, each with a duration of 15 min.

### 2.2. Descriptive Aspects of the Acoustic Environments

Keynote sounds were used to analyze some descriptive aspects of the acoustic environments. This concept refers to sounds that are continually heard or that have a constant presence strong in the acoustic environment [59]. To identify the keynote sounds of the eight places, the subjective test included a list of sounds, and participants were asked to indicate how dominant they perceived each one. The response options were “non-dominant”, “little dominant”, “moderately dominant”, “very dominant” and “totally dominant” and each option corresponds to values of −1, −0.5, 0, 0.5 and 1 respectively.

Although the objective of this work focuses on deepening the study of contextual and semiotic aspects in management and planning processes of urban soundscapes, it was considered relevant to include an acoustic indicator to complement descriptive aspects of the acoustic environments. In this study, the equivalent continuous A-weighted sound pressure level L_Aeq_ was used, since it is a widely sound descriptor and shows good correlations with perceptual attributes as pleasant and comfort [21,24,57,60,61].

### 2.3. Subjective Evaluation

**Figure 2:**
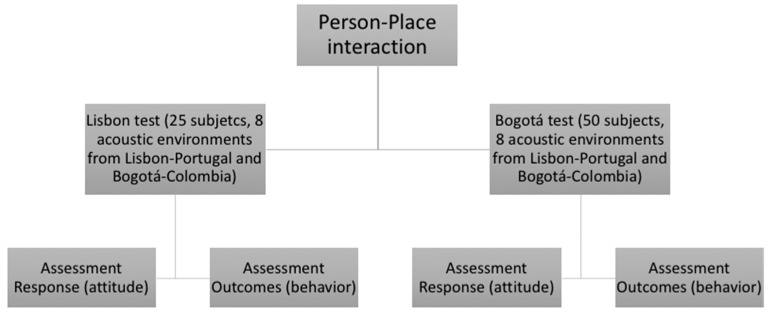
General design of the subjective evaluation test.

The work focuses on the analysis of the influence of the *Person-Place Interaction* with input variables such as responses and outcomes. For this purpose, a test was applied where listeners evaluated unknown sites and places with which they had interacted. Figure 2 describes the general design of the subjective tests.

The laboratory tests were attended by 75 listeners (25 in Lisbon and 50 in Bogotá), in line with other similar previous works [62,63], all aged between 18 and 60 years old. The participants knew the places of their respective cities and had advanced knowledge in the acoustics field (sound engineering students and professors from Universidad de San Buenaventura-Bogotá and students and professors of the architecture and acoustics from Instituto Superior Técnico, University of Lisbon). No distinction is made between experts and non-experts because the interaction of listeners with acoustic environments can generate a natural process of training of the listeners mediated by context [46]. All participants contributed voluntarily to the test and were informed in writing and orally about the research’s objectives and methodology. They also authorized the use of the collected information for strictly academic purposes.

**Figure 3:**
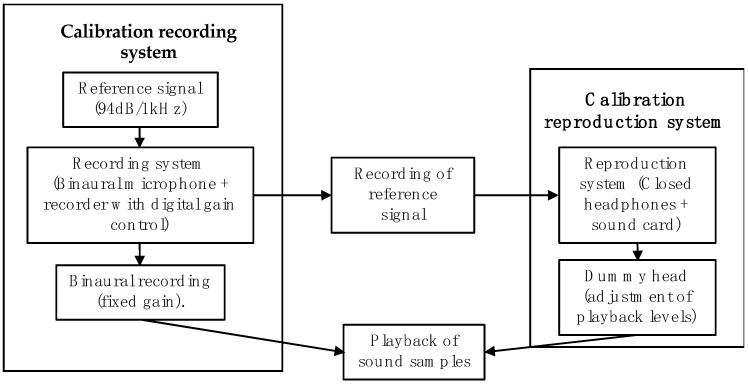
Calibration process diagram for the recording and reproduction of acoustic environments in laboratory test.

From the fifteen minutes of the binaural recording obtained in each location, thirty seconds of audio for each urban acoustic environment in study were selected. For the audio edition, the information obtained in the previous visits and sound walks to each place was considered, trying that the audio samples represented some of the observed activities. Eight audio fragments of 30 seconds duration corresponding to the sites were then presented to each listener, with a maximum total duration of 15 min. The time length of each audio fragment was chosen taking into account previously works (which presented audios between 6 seconds and 1 min [20,21,38,46,64,65,66,67,68]) and sound quality recommendations (tests were designed of 30 min maximum concerning the importance of not tiring the listener [62,63,69]). The playback order was randomized for each tested subject to avoid bias by playing order [63]. The listeners played each sample at a time and responded to the different questions of the test according to the self-paced methodology. The separation between each audio fragment varied between one minute and one minute and a half depending on the speed of response of the participants. Headphones were used by the listeners. Both the recording and the playback systems were duly calibrated, using an artificial head to ensure that the sound pressure levels presented to the listeners were as close as possible to those they would perceive at the recording location (see Figure 3). The tests were carried out in the recording studios of the Universidad San Buenaventura, Bogotá and in the anechoic chamber of the Instituto Superior Técnico (IST), Lisbon, University of Lisbon.

The subjective test was divided into two parts, the first related to the responses (attitudes) and the second related to the outcomes (behaviors) according to the model depicted in the depicted in the ISO 12913-1 standard. For the assessment of responses Axelsson’s model was used, considering the eight perceptual attributes: *pleasant*, *chaotic*, *exciting*, *uneventful*, *calm*, *annoying*, *eventful*, and *monotonous* [21,70,71]. These attributes allow the estimation of the main components of the ortho-normalized bidimensional model: *Pleasantness* and *Eventfulness* [71]. For the assessment of each attribute the technique of the semantic response scale (response scale) was followed asking the subjects “to what extent do you consider that the following eight factors describe the sound environment heard?”. Each attribute had five levels of response: fully agree, partially agree, disagree, partially disagree, and strongly disagree. For the data processing, each of these levels took values between +1 to a step of 0.5, being +1 completely agree and −1 totally disagree. Considering that the participants’ native language was Spanish and Portuguese, the Axelsson attributes were translated from English into Spanish and Portuguese. In the translation process, the eight attributes were considered as related (orthogonal model), so the words used in the test were the product of the combination of the two main components (Pleasantness and Eventfulness).

Concerning the outcomes (behaviors), this study deepened in the decisions that the listeners would take in a site with an acoustic environment like was presented in the audio sample. For this, questions related to the *Permanency Time* and the *Possible Use* were established. The permanency times were presented by ranges (less than 10 min, between 10 and 30 min, between 30 and 60 min, between 1 and 2 h, and more than two hours), while the possible uses were presented from a list of activities (physical activity, place of transit, contemplation, reading and meditation, general rest, and other activities).

For the data obtained from responses (attitudes), Shapiro-Wilk’s normality tests were applied, finding that the data do not present a normal distribution. In the comparative study between Lisbon and Bogotá listeners, the data were analyzed on the basis of the U Mann-Whitney tests for numerical data (responses resulting from the evaluation of perceptual attributes) and contingency tables for nominal data (in the case of questions related to outcomes), where the Pearson’s statistical significance Chi-square and the likelihood-ratio chi-square tests were carried out.

Finally, from the results obtained from the responses and outputs of the evaluators, a discussion is proposed in the light of the anthropological concept of “non-places” [72], as well as semiotic aspects (Use-Meaning relationship) and models of semiosis based on the Peirce model [50].

## 3. Results 

### 3.1. Keynote Sounds and Sound Pressure Levels

Table 1 presents information on the keynote sounds and the L_Aeq_ of the different places under study. Regarding the keynote sounds of the eight places, both Lisbon and Bogota evaluators determined that the keynote sounds are road traffic for the BOAPA and BOC197 places, sounds of nature for LJFG and BOPSB, the human voice for LJES and LJPR, and the water source for the BOFAET. Only BOPL presented differences for the keynote sounds between the evaluators of Bogota and Lisbon, where the panel of Bogotá considered that the keynote sound of this space is speech, while the panel of Lisbon considered that the keynote sound of this space it’s road traffic.

Regarding the sound pressure levels, the table shows that those with higher levels are BOFAET (75.4 dBA and keynote sound water) and BOC197 (74.6 dBA and keynote sound road traffic), while the lowest levels are from the BOPSB (51.4 dBA and keynote sound of nature) and LJPR (56.6 dBA and keynote sound of speech). It can be seen that the higher levels do not correspond only to places with keynote sound of road traffic, as well as the places with the lowest L_Aeq_ does not correspond only to the keynote sound of nature.

### 3.2. Listeners’ Responses

#### Differences between Lisbon and Bogotá Responses

**Figure 4:**
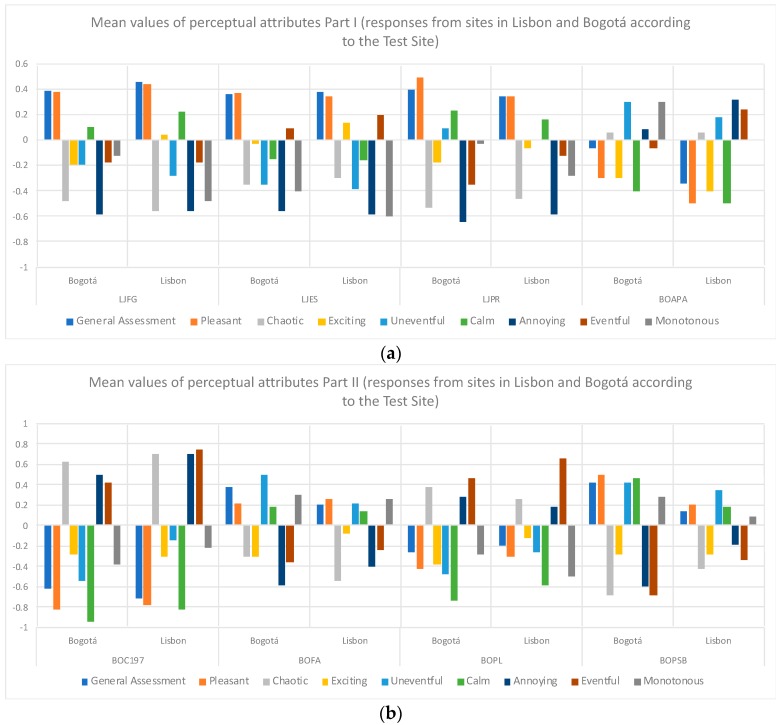
(**a**) Mean values of perceptual attributes Part I (responses from sites in Lisbon and Bogotá according to the listener’s Test Site); (**b**) Mean values of perceptual attributes Part II (responses from sites in Lisbon and Bogotá according to the listener’s Test Site).

Figure 4a,b present the results obtained for the eight attributes of the Axelsson model, as well as for the general assessment according to the place of origin of the evaluators. It is possible to appreciate differences according to the listener’s Test Site for attributes such as Exciting, Monotonous, Eventful, Annoying, among others, although to determine if these differences are statistically significant it is necessary to apply the U Mann-Whitney nonparametric test. Table 2 presents these results.

For the LJFG site, statistically significant differences were found only for the *Exciting* and *Monotonous* attributes and according to the mean rank values: the Lisbon listeners considered this place more Exciting, while evaluators from Colombia rated it as Monotonous. The LJES site did not show statistically significant differences in any perceptual attribute, while the LJPR site showed statistically significant difference only in the *Monotonous* attribute (judged as more *Monotonous* in Bogotá than in Lisbon).

Regarding the Colombian sites, the BOAPA presents statistically significant differences in the *General Assessment* and in the *Eventful* attribute (a better General Assessment was found in Bogotá though it was judged more Agitated by the Lisbon listeners). The BOC197 site presented statistically significant differences in the *Uneventful*, *Calm*, *Annoying*, and *Eventful* attributes (higher mean rank values were found in the evaluations in Lisbon for the four attributes), while the sites BOFA and BOPL did not present statistically significant differences in none of their attributes. Finally, the BOPSB site was the one with the highest number of attributes with statistically significant differences in *The General Assessment*, *Pleasant*, *Chaotic*, *Annoying*, and *Eventful* (mean rank values in *General assessment* and *Pleasant* were found by Bogotá listeners, while higher mean ranks values were found in *Chaotic*, *Annoying*, and *Eventful* by Lisbon listeners).

**Figure 5:**
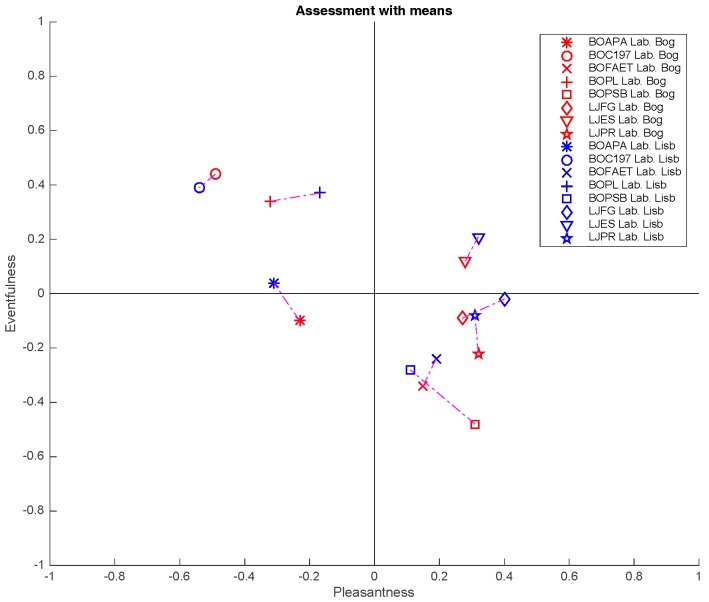
Application of Axelsson’s two-dimensional model. In red, places evaluated in Bogotá-Colombia. In blue, places evaluated in Lisbon-Portugal.

Figure 5 presents the Cartesian representation of the Axelsson’s model according to the evaluations carried out in Lisbon and Bogotá. One can see that, although small shifts are found in the *x* and *y* axes for the different places under study according to their Test Site, the shifts are not considerable. In fact, only one of the eight places under study (BOAPA) showed a slight change of quadrant, represented in the change of the main component Eventfulness.

### 3.3. Outcomes: Permanency Time and Possible Uses

**Figure 6:**
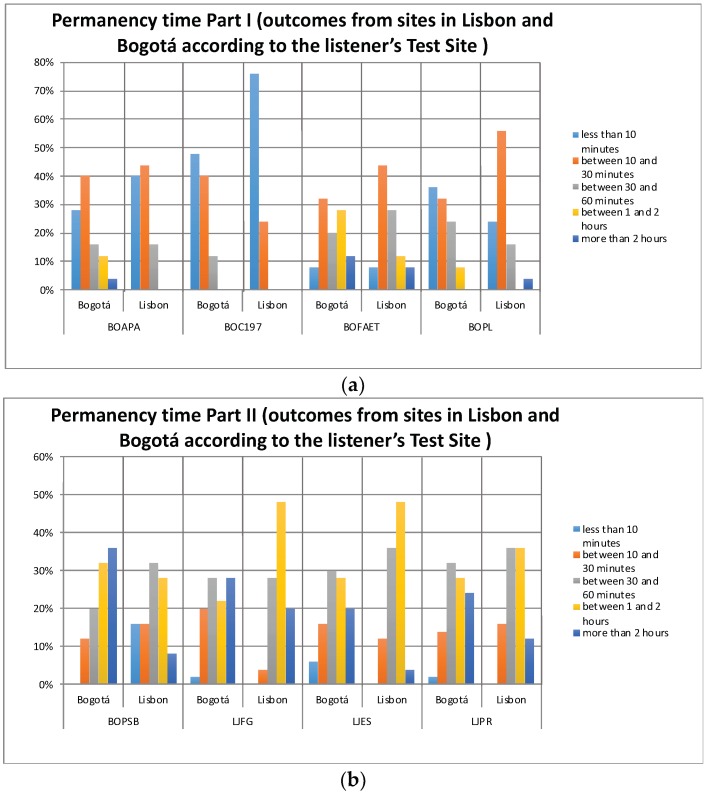
(**a**) Outcomes: permanency time of the listener according to test sites in Lisbon and Bogotá places (Part I); (**b**) Outcomes: permanency time of the listener according to test sites in Lisbon and Bogotá places (Part II).

**Figure 7:**
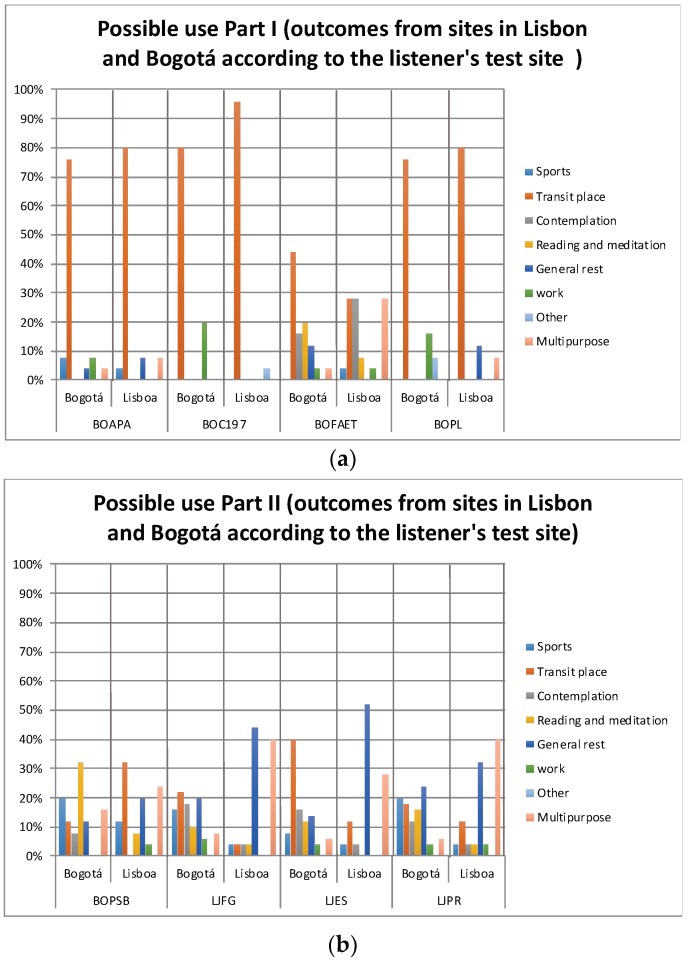
(**a**) Possible use that might be given by the evaluators in places with acoustic environments such as the ones presented in the recordings (Part I); (**b**) Possible use that might be given by the evaluators in places with acoustic environments such as the ones presented in the recordings (Part II).

Figure 6a,b and Figure 7a,b show the outcomes of the evaluators, represented in the hypothetic *Permanency Time* and the *Possible Uses* of Bogotá and Lisbon listeners in sites with acoustic environments such as those that are presented in the recordings. There are some notable differences according to the place of origin of the listener in the outcome *Permanency Time*, as in BOC197 where for the seventy-six percent of the listeners in Lisbon it would only be less than 10 min, compared to the forty-nine percent of listeners from Bogotá that would be that same amount of time. However, in BOAPA or LJPR these differences are not so simple to appreciate. Regarding the *Possible Uses*, the LJFG results reveal differences between the listeners in Lisbon and in Bogotá, but similarly to the variable Permanency Time, in places such as BOAPA or BOFAET differences are not easily appreciated regarding the listener’s place of origin. To determine whether these differences are statistically significant, the Pearson’s statistical significance Chi-square and the likelihood-ratio Chi-square tests were applied. The results are depicted in Table 3.

Regarding the *Permanency Time*, of the eight test sites under study, only two revealed a statistically significant association between the listener’s Test Site and this variable in particular. Evaluators from Bogotá would stay longer in the BOC197 and BOPSB sites than Lisbon evaluators.

As for the *Possible Use*, five of the eight sites under study showed a statistically significant association between this variable and the listener’s Test Site. It is interesting to note that of the three sites that did not show a statistically significant association between the variables listener’s Test Site and Possible Uses, two can be considered common places in any city (a water fountain in BOFAET and a BOAPA highway), while the third one, the BOPSB park, does not present an association between these variables, though as mentioned before, this site shows an association between the variables of *Permanency Time* and listener’s Test Site.

Carrying on with the analysis of the variables *Possible Use* and Listener’s *Test Site* variables, the results allow the appreciation of a second group of sites (the Lisbon LJFG, LJES, and LJPR places, and the BOC197 and BOPL Bogotá places), which do present statistically significant association between the variables Test Site and Possible Use. Although the BOPL site has the highest percentages for Transit Site within the variable Possible Use for the two evaluation sites (76% in Bogotá and 80% in Lisbon), evaluators from Bogotá would also use this site for Work, while evaluators from Lisbon would use it for General Rest. The same phenomenon was also found for the BOC197 site. In the LJFG site, strong differences were found, given that responses of the Lisbon listeners showing that this site would be used mainly as a place of General Rest and a Multipurpose place, while in Bogotá the main percentages were scattered between Place of Transit, General Rest, and Contemplation. Similar results were found for the LJES site, where the evaluations of Lisbon show the use to be mainly related to General Rest, while once again in Bogotá it would be used mainly as a Site of Transit. Finally, the LJPR site for the evaluators of Lisbon would be used mostly as a Multipurpose place and General Resting place, while in Bogotá it would be used as a place of Transit and a General Resting site as well.

## 4. Discussion

The discussion herein is based on descriptive aspects of the acoustic environments, Person-Place interaction and contextual as well as semiotic aspects. The results show that the listeners of the two countries identified the same keynote sounds for seven out of the eight places under study. This may mean that, for the cases under study, the possibility of identifying the keynote sound is not affected by the Person-Place relationship. However, it is interesting that although no differences were found in this descriptive aspect, some differences in responses and outcomes were found according to the place of origin of the listener. This may indicate that one thing is the ability to detect and identify the keynote sound of the acoustic environments and another is the meaning that is given to them by the listeners (meaning that is mediated by the context).

Likewise, while it is clear that high levels of L_Aeq_ affect people’s health (which implies that a first step for urban acoustic management is the control and reduction of these sound levels), not necessarily low energy levels may ensure a longer permanency time in places, or a specific use (outcomes). For example, BOFAET presented the highest values of L_Aeq_, but it was a place where most people would be between 10 and 30 min. However, the places with higher L_Aeq_ if they were considered more annoying by the listeners what agrees with previous findings, where parameters associated with energy levels influence the degree of comfort and/or discomfort [21,25,57,61,73].

Moreover, three out of the eight sites under test presented statistically significant differences for the variable *Responses* (perceptual attributes) according to the listener’s *Test Site* in the *Eventful* perceptual attribute, while the *Monotonous*, *Calm*, and *Unpleasant* attributes presented significant differences in only two sites. The general assessment also presented differences in two places, while attributes such as *Pleasant*, *Chaotic*, *Exciting* and *Uneventful* only showed statistically significant differences in one of the sites under study. As previously discussed, these eight components directly impact the estimation of the main components Eventfulness and Pleasantness of Axelsson’s model. Therefore, if there are few associations between the listeners’ Test Site and the perceptual attributes, there will be little impact on the cartesian representation by quadrants on this model.

Since the variable *Test Site* implies a previous knowledge of the places by the people involved in the experiments, there is, consequently, an interaction between Person and Test Site (activities and characteristics), hence it can be said that, under the conditions of these tests, there is a relation between the *responses* (attitudinal aspects) and the *Person-Place Interaction*. Therefore, from previous knowledge and experiences in places, it is possible to generate differences in listeners’ responses (e.g., for the LJFG place, Lisbon listeners considered it as more Exciting, while evaluators from Colombia rated it as Monotonous).

Regarding the *Outcomes* (Permanency Time and Possible use) and place of testing, it was found that five out of the eight sites under study had an association between the variables *Test Site* and the *Possible Uses*, although only two were dependent on the variables *listener’s Test Site* and *Permanency Time*. These results show that, under the conditions proposed during the development of this experiment, the outcomes (or behaviors) have a dependency according to the Person-Place interaction. To better understand these results, the semiotics approach seems useful, where sites, usage, sound, and meaning have a direct relationship.

The meaning of the places depends on the use given to the places (meaning = use) [74], which is why users are able to read a place and assign it a meaning. Therefore, a place can be considered a “center of meaning” that feeds on the experiences and attachments of the people [75]. For this reason, it is not possible that representative places of each city (such as Lisbon and Bogotá parks) can have the same use and consequently the same meaning for evaluators of different countries, when the evaluators do not have previous knowledge, or have not used these places or do not have the same practices and activities. In view of the above, although it is true that the recordings of the acoustic environment provide an idea of the activities and practices developed on the place, the recordings do not yield information related to the use of the place according to the context, conveying information from the practices or activities that are common. The *Person-Place Interaction* is the product of the direct experience of the person, which generates memories and previous background that raise the expectations about the activities to be carried out in such place. Under laboratory conditions and without such previous experiences, the listeners will associate the acoustic environments with experiences of their own, with places of different characteristics, which might generate a difference of “meanings” according to the place of origin of the listener.

Likewise, it is interesting to see the emergence of sites that can be considered common or mainstream to any city (a highway or a water fountain, for example), since there is no association between the variables *Possible Use* and the *Listener’s Test Sites*, which means that its “meaning” is the same regardless of the Listener’s Test Site. It is here that the concept of “*the non-place*” arises and becomes relevant. A place can be defined as “an identity, relational and historical site” so that a space that “can not be defined either as an identity, relational or historical site is considered a non-place” [72]. Bringing this concept to the soundscape field, acoustic environments that do not represent or generate identity, evoke a memory or awake some sense of belonging in the listener can be considered as *sonorous non-places*. To delve into this concept, two out of the eight cases presented in this study will be shown: the BOAPA site (north highway of Bogotá) and LJES site (urban park of the city of Lisbon).

The BOAPA site is the main highway of Bogotá and it communicates the Colombian capital with the north of the country. It crosses part of the city in south-north and north-south directions with around 14,000 vehicles transiting through it every hour (both directions are considered for this estimation). It should be noted, as a main feature of this specific highway, that part of its layout has exclusive lanes where biarticulated buses belonging to the Bogotá mass transportation system circulate. Summarizing, it is a common highway that, although located in Bogotá, Colombia, could also be anywhere else in the world. Its use is just like that of any other highway: a main road where vehicles circulate. It could be the perfect example of a *sonorous non-place*, given that the sound sources that compose it and the uses that are given to this space do not vary significantly depending on the city. When the association tests for the variables *Possible Use* and *Listener’s Test Site* were applied, it was found that the use that would be given to an acoustic environment like the one presented in the recording, would be the Place of Transit both for the evaluators of Lisbon and for those of Bogotá. This implies that for the two groups of evaluators the place has the same use and therefore the same meaning, which exemplifies a sonorous non-place.

Conversely, the LJES park is located in the Lapa neighborhood of the city of Lisbon, built at the end of the 19th century. Its 4.6 hectares feature two lakes, children playgrounds, kiosks and equipment for physical exercise. In the lakes one can see ducks, swans and geese, and prowling around the garden a majestic peacock can sometimes be spotted. There is a kiosk that is usually used for philharmonic concert and overall enjoyment. One of its streets adjoins the Basilica da Estrela in Lisbon, a must see for tourists who can also get to the place on the legendary E28 wooden tram. In the summertime, concerts and picnics are common for tourists and residents. The binaural recordings presented to the evaluators registered the sound of the bells of the Estrela Basilica, the sounds of birds and nature, as well as the sound of public transportation. When the results for the variables *Possible Use* and *Listener’s Test Site* were analyzed, it was found that they have a statistically significant association: while the people from Bogotá would identify it mostly as a Place of Transit, people from Lisbon would identify it as a General Resting place. Therefore, the results suggest in this case that the history and the direct contact with the place impact the perceived “meaning” and, due to the decontextualization of the evaluators of Bogotá, and the multiple “meanings” that can be assigned by the people of Lisbon, such “meanings” are very different depending on the listener’s Test Site.

### Contextual and Semiotic Aspects Applied to Urban Soundscapes Management, Design and Planning Processes

The cities are a reflex of their inhabitants and their culture, and thus different forms of expression can be found, represented by different languages. Therefore, every action potentially becomes a sign that will be deciphered by the people according to their repertoire and context. In this sense, the city speaks, transmits information [76], and the sonorous language is a powerful carrier of meaning. Since the acoustic environments are the product of practices and activities developed by people, the soundscape concept constitutes another approach for relating and understanding the city. The aim thus, in processes of design, planning, and management of urban spaces, consists of designing and implementing urban acoustic environments that carry coherent information to the people in order that they assign a meaning to the signs according to space.

Likewise, if a place acquires “meaning” according to its use, the “meaning” of the acoustic environment will be influenced by the *Person-Place Interaction* (contextual aspects). When performing an inverse exercise in design, planning and management processes, it is worth asking: what is the “Use-Meaning” of the place? This exercise involves the understanding of the semiotic process where three aspects, *Place*, *Acoustic Environment* and *Soundscape*, are related to the context to generate a “meaning”, that is, it implies a semiosis model applied to urban soundscape.

**Figure 8:**
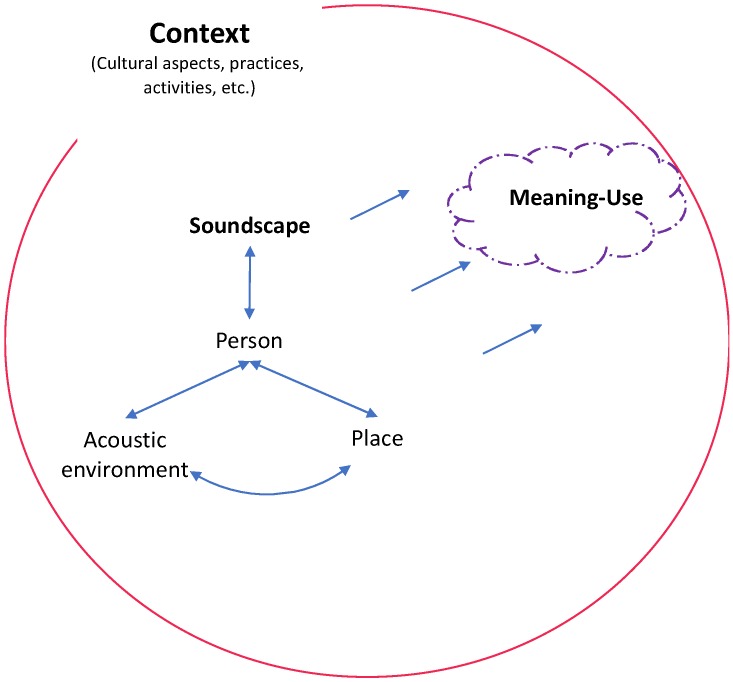
Semiosis model for urban soundscapes.

Considering the concept of semiosis as a process of selection, organization, coordination, and structuring of the items of perception and objects of experience [48], Figure 8 depicts the core of a semiosis model adjusted for soundscapes. The model’s purpose is fed by previous findings in the fields of sound quality, semiotics, and soundscape design [15,48,49,50,77], where the *Place*, the *Acoustic Environment* and the *Soundscape* are related, with *the Person* as the central axis. The semiosis model is based on the general triadic model proposed by Peirce, which was adapted by Jekosch for sound quality processes. In this adaptation, the acoustic environment is a sign carrier that represents a specific place (object), the soundscape is the result of the cognitive process developed by a person, which acquires meaning from the context (which in turn influences the city, the persons and the acoustic environments).

The process presented in Figure 8, establishes a connection between the Place, the Acoustic environment and the resulting Soundscape, and go in the same line of previous works, that indicate the need to relate the use of the spaces to the soundscapes [43,77]. Persons interact with places, so the acoustic environment is configured from the *affordance* place, the activities and practices developed. The soundscape is, therefore, a product of the interaction of Person with their Place and their Acoustic environment, which in turn feeds on the context and contributes to the meaning of places.

Based on the fact that the soundscape management approach broadens traditional acoustic management, due to the fact that its main objective is the well-being of people, this work deepens the need to manage soundscapes that improve the environmental experience of people (according to the place’s use, and the desired responses and outcomes) and contribute to the strengthening of the meaning of places.

The soundscape design process can thus be deepened by putting forward questions related to the contextual and semiotic aspects. Their answers may provide input for the criteria of evaluation, design, management, and planning of acoustic environments, according to Bento-Coelho’s soundscape design roadmap (Table 4) [77]:

## 5. Conclusions

The recent developments on the concept of soundscape as a needed alternative in the analysis, assessment, and design of urban acoustic environments has led to new challenges, since an interdisciplinary approach is required that broadens the perspective of the environmental acoustic management. Specifically, in this work aspects related to *Person-Place Interaction* and its impact on people’s responses and outcomes were studied, building a discussion based on concepts from the fields of psychology, anthropology, and semiotics.

In this study, the laboratory tests showed that the *responses* (perceptual attributes indicating attitudes) did not generate considerable associations with respect to the Listener’s Test Site (*Person-Place Interaction*). However, the *outcomes* (associated with the behavior = possible use) revealed associations with the Listener’s Test Site, generating two effects: (1) the association of the “place meaning” to the Possible Use and (2) the emergence of a *sonorous non-place*.

The association of the Place Meaning to the Possible Use, as well as the relation of these aspects with the soundscapes, prove the need to carry out studies with multidisciplinary approaches in this field. In this work, in addition to considering aspects of ecological perception, acoustic environments were analyzed as carriers of meaning, which acquire meaning from contextual aspects. Under the conditions of this study, it was established that for the formation of meaning of an acoustic environment the previous experiences of the people, the activities for which that space was created, and the cultural and spatial aspects that influence the practices developed in the places must be taken into consideration. That is, the *Person-Place Interaction* impacts the meaning of acoustic environments from its use.

Likewise, the homogenization of places also impacts soundscapes, and if there are similar sound sources and similar site uses, the soundscapes will also be similar. This leads to the consideration of *sonorous non-places*, places that have no difference in their “meaning” independently of the *Person-Place Interaction*. Of course, it is not possible or necessary to think that all places must have a unique identity, although it is important to preserve the spaces that require so.

Therefore, in processes of design, planning, and management of environmental acoustics, not only taxonomic, energetic, temporal, and spatial analysis of the acoustic environments are necessary, but a contextual and semiotic study of urban space is also required. In this sense, the analysis of the use of the place, of the people who will use the space, of the coherence between the space and the acoustic environment and the desired meaning, are key to the success of the project. When considering the soundscape as part of a semiosis process, it is important to emphasize in the interaction of the triad *Place*, *Acoustic environment* and *Soundscape*, the *Person* as the central axis.

This work’s results seem to suggest the need to continue expanding the processes of management, design, and planning of urban soundscapes, by considering contextual and semiotic aspects that allow a better understanding of the environmental experience and strengthen the meaning of the places, so as to further ensure the people’s well-being. In this way, the questions opened up in this work can complement the traditional approaches and enrich the acoustic design and management processes. These questions together with the proposed semiosis model can contribute concretely to create inputs required for such processes.

Finally, urban acoustic designers have the responsibility to present coherent information between the acoustic environment and the use of the place. This, in turn, implies defining multidisciplinary strategies that establish how and when information is generated, to help in the process of formation of meaning of the places by the listeners.

## Figures and Tables

**Table 1:** Keynote sound and L_Aeq_ of the acoustic environments in study.

Place	City	Keynote Sound	L_Aeq_ (dBA)
LJES	Bogotá	Speech (mean value 0.57 and 88% of detection)	65.6
	Lisbon	Speech (mean value 0.54 and 84% of detection)	
LJFG	Bogotá	Nature (mean value 0.43 and 80% of detection)	58.4
	Lisbon	Nature (mean value 0.46 and 76% of detection)	
LJPR	Bogotá	Speech (mean value 0.49 and 78% of detection)	56.6
	Lisbon	Speech (mean value 0.38 and 60% of detection)	
BOAPA	Bogotá	Road Traffic (mean value 0.66 and 96% of detection)	63.9
	Lisbon	Road Traffic (mean value 0.70 and 96% of detection)	
BOC197	Bogotá	Road Traffic (mean value 0.94 and 100% of detection)	74.6
	Lisbon	Road Traffic (mean value 0.94 and 100% of detection)	
BOFAET	Bogotá	Water (mean value 0.80 and 93% of detection)	75.4
	Lisbon	Water (mean value 0.90 and 100% of detection)	
BOPL	Bogotá	Speech (mean value 0.56 and 88% of detection)	68.7
	Lisbon	Road Traffic (mean value 0.50 and 92% of detection)	
BOPSB	Bogotá	Nature (mean value 0.50 and 80% of detection)	51.4
	Lisbon	Nature (mean value 0.42 and 68% of detection)	

**Table 2:** Results of the U Mann-Whitney test with *α* = 0.05 for evaluators from Bogotá and from Lisbon. Green values represent statistically significant differences in the responses according to listener origin. Shaded values represent the highest average rank value between listeners in Bogotá and Lisbon.

Place	Statistics	General Assessment	Pleasant	Chaotic	Exciting	Uneventful	Calm	Annoying	Eventful	Monotonous
LJFG	U	540.0	537.0	577.5	437.5	577.5	535.5	620.0	625.0	338.0
	*p* value	0.282	0.286	0.571	0.024	0.575	0.281	0.952	1.000	0.001
	Mean Rank Bogotá	36.30	36.24	38.95	34.25	38.95	36.21	37.90	38.00	43.74
	Mean Rank Lisbon	41.40	41.52	36.10	45.50	36.10	41.58	38.20	38.00	26.52
LJES	U	597.0	624.5	594.5	497.0	612.5	615.0	622.5	570.0	499.5
	*p* value	0.731	0.995	0.722	0.126	0.882	0.906	0.976	0.504	0.128
	Mean Rank Bogotá	37.44	38.01	37.39	35.44	38.25	38.20	38.05	36.90	40.51
	Mean Rank Lisbon	39.12	37.98	39.22	43.12	37.50	37.60	37.90	40.20	32.98
LJPR	U	569.0	500.5	530.0	542.0	569.5	550.0	543.0	469.0	433.5
	*p* value	0.474	0.137	0.258	0.324	0.517	0.375	0.319	0.068	0.025
	Mean Rank Bogotá	39.12	40.49	36.10	36.34	39.11	39.50	36.36	34.88	41.83
	Mean Rank Lisbon	35.76	33.02	41.80	41.32	35.78	35.00	41.28	44.24	30.34
BOAPA	U	207.5	258.0	309.5	278.5	271.5	293.0	237.0	212.0	218.0
	*p* value	0.030	0.265	0.951	0.489	0.395	0.690	0.127	0.040	0.058
	Mean Rank Bogotá	29.70	27.68	25.62	26.86	27.14	26.28	22.48	21.48	29.28
	Mean Rank Lisbon	21.30	23.32	25.38	24.14	23.86	24.72	28.52	29.52	21.72
BOC197	U	278.5	280.5	302.5	306.5	193.5	229.5	222.5	204.5	256.5
	*p* value	0.466	0.463	0.830	0.904	0.017	0.025	0.055	0.022	0.261
	Mean Rank Bogotá	26.86	24.22	25.10	25.74	20.74	22.18	21.90	21.18	23.26
	Mean Rank Lisbon	24.14	26.78	25.90	25.26	30.26	28.82	29.10	29.82	27.74
BOFA	U	264.0	288.5	244.5	243.0	243.5	285.0	247.0	264.5	303.5
	*p* value	0.317	0.613	0.168	0.156	0.160	0.580	0.165	0.332	0.856
	Mean Rank Bogotá	27.44	24.54	28.22	22.72	28.26	26.60	22.88	23.58	25.86
	Mean Rank Lisbon	23.56	26.46	22.78	28.28	22.74	24.40	28.12	27.42	25.14
BOPL	U	286.5	269.5	268.5	229.5	242.5	236.5	276.5	230.5	242.5
	*p* value	0.595	0.375	0.371	0.095	0.151	0.100	0.467	0.075	0.154
	Mean Rank Bogotá	24.46	23.78	27.26	22.18	22.70	22.46	26.94	22.22	28.3
	Mean Rank Lisbon	26.54	27.22	23.74	28.82	28.30	28.54	24.06	28.78	22.70
BOPSB	U	208.5	213.5	203.5	305.5	263.5	218.5	196.0	183.0	233.0
	*p* value	0.034	0.045	0.025	0.887	0.314	0.057	0.019	0.008	0.108
	Mean Rank Bogotá	29.66	29.46	21.14	25.22	27.46	29.26	20.84	20.32	28.68
	Mean Rank Lisbon	21.34	21.54	29.86	25.78	23.54	21.74	30.16	30.68	22.32

**Table 3:** *X*^2^ and the likelihood-ratio tests with a *α* = 0.05 for the eight soundscapes for both the evaluators from Bogotá and from Lisbon. Green values represent statistically significant association between the outcomes and the listener origin.

Test Site	Permanency Time						Possible Use
	Statistics	Value	df	*p* Value (2-Sided)	Value	df	*p* Value (2-Sided)
BOAPA	*X* ^2^	4.577	4	0.376	3.026(a)	4	0.77
	Likelihood Ratio	6.125	4	0.301	3.818	4	0.77
BOC197	*X* ^2^	5.581	2	0.056	6.364(a)	2	0.05
	Likelihood Ratio	6.764	2	0.044	8.682	2	0.05
BOFA	*X* ^2^	2.607	4	0.643	11.493(a)	6	0.05
	Likelihood Ratio	2.658	4	0.658	13.66	6	0.051
BOPL	*X* ^2^	5.636	4	0.198	11.026(a)	4	0.008
	Likelihood Ratio	6.823	4	0.181	15.275	4	0.006
BOPSB	*X* ^2^	9.356	4	0.049	10.273(a)	6	0.094
	Likelihood Ratio	11.272	4	0.038	11.785	6	0.093
LJFG	*X* ^2^	7.504	4	0.096	22.646(a)	6	0
	Likelihood Ratio	8.3	4	0.086	24.59	6	0.001
LJES	*X* ^2^	6.701	4	0.155	25.736(a)	6	0
	Likelihood Ratio	8.239	4	0.11	28.267	6	0
LJPR	*X* ^2^	2.173	4	0.773	17.942(a)	6	0.004
	Likelihood Ratio	2.584	4	0.74	18.474	6	0.009

**Table 4:** Contextual and semiotic aspects in the Bento Coelho’s soundscape design roadmap.

*Define purpose and activities* What is the desired objective and/or meaning of the place?
*Define acoustic objectives according to purpose and activities* How does the acoustic environment contribute to the environmental experience and/or the meaning of the place? What is the Person-Place relationship? (including expectations, use, and appropriation of people)?
*Identify listening places and listening itineraries* What activities take place in the places? (according to the time of year, hour, etc.)
*Identify sound sources and sound components* Which sounds identify or represent the most characteristic activities of the place? Which sounds enhance the environmental experience and are coherent with the context, the meaning and use of the place?
*Identify sound propagation paths* How do sounds and sound sources interact with their environment?
*Identify preferred and unwanted preferred and unwanted sounds* Which sounds do not favor the environmental experience, are incoherent with the context, the meaning and use of the place?
*Manage sound components (Diminish unwanted sounds, enhance preferred sounds, and identify wanted sounds in context)* How to enhance and/or create sounds that favor the environmental experience, the use and meaning of the place? How to reduce sounds that do not favor environmental experience, to the detriment of the use and meaning of the place?
